# Succinic Acid Improves the Metabolism of High-Fat Diet-Induced Mice and Promotes White Adipose Browning

**DOI:** 10.3390/nu16223828

**Published:** 2024-11-08

**Authors:** Yuxuan Yang, Liang Luo, Yiqi Li, Xiangda Shi, Chen Li, Jin Chai, Siwen Jiang, Rong Zheng

**Affiliations:** 1 Agricultural Ministry Key Laboratory of Swine Breeding and Genetics & Key Laboratory of Agricultural Animal Genetics, Breeding, and Reproduction of Ministry of Education, Huazhong Agricultural University, Wuhan 430070, China; yangyuxuan418@webmail.hzau.edu.cn (Y.Y.); luoliang0723@163.com (L.L.); 13357684267@163.com (Y.L.); 17866705361@163.com (X.S.); lichen14@webmail.hzau.edu.cn (C.L.); chaijin@mail.hzau.edu.cn (J.C.); jiangsiwen@mail.hzau.edu.cn (S.J.); 2 The Cooperative Innovation Center for Sustainable Pig Production, Wuhan 430070, China

**Keywords:** succinic acid, obesity, lipid metabolism, insulin resistance, fat browning

## Abstract

Succinic acid plays a crucial role as an essential intermediate in the mitochondrial tricarboxylic acid cycle in mitochondria. In recent years, growing evidence has supported the the important role of succinic acid in fat metabolism. Therefore, we aimed to investigate the effects of succinic acid on adipose tissue metabolism and insulin sensitivity in high-fat diet (HFD)-induced obese mice and try to explore its potential mechanism. We found that the addition of succinic acid (40 mM) to drinking water inhibited the hypertrophy of inguinal white adipose tissue (iWAT) in HFD-induced mice. Furthermore, succinic acid supplementation enhanced insulin sensitivity and improved their glucose tolerance in obese mice. Interestingly, succinic acid supplementation improved lipid metabolism in HFD-fed mice, as shown by decreased serum levels of TG, TC, LDL-C, and increased HDL-C. In addition, succinic acid supplementation increased the expression of browning markers and mitochondria-related genes in iWAT. Further studies showed that the addition of succinic acid to drinking water promotes the browning of iWAT by activating the PI3K-AKT/MAPK signaling pathway. These results suggest that succinic acid has the potential to be used as an effective component for dietary intervention and may, therefore, play an important role in ameliorating and preventing obesity and associated metabolic diseases caused by HFD.

## 1. Introduction

As a result of the substantial increase in overall productivity levels, the accessibility of food has significantly improved, thereby diminishing the prevalence of malnutrition and mitigating the threat of famine for the majority of individuals. The subsequent consequence has been the rapid escalation and prevalence of obesity, emerging as a pressing and pervasive global health concern in contemporary society [1,2,3]. The condition of obesity is correlated with a range of metabolic disorders, including type II diabetes, non-alcoholic fatty liver disease, and an increased susceptibility to cancer [4,5,6]. The current market offers a variety of pharmacological interventions and therapeutic approaches for obesity; however, the majority of them are associated with significant adverse effects, such as gastrointestinal disturbances (diarrhea, nausea) and potential rebound effects [7,8]. The urgent need for the development of efficacious and safe management strategies to prevent or treat obesity is evident [9,10].

The accumulation of triglycerides as lipid droplets in adipose tissue is the underlying mechanism contributing to obesity [11,12]. Adipose tissue can be categorized into white adipose tissue (WAT), beige adipose tissue, and brown adipose tissue (BAT) based on morphology and functionality [13,14]. The primary function of WAT is to serve as depots for fat storage [15,16]. The activation of BAT triggers the release of energy in the form of heat, which is dependent on oxidative phosphorylation to decouple ATP synthesis from Uncoupling Protein 1 (UCP1) [17]. The expression of UCP1 can also be detected in WAT, thereby inducing the browning of white adipose [18,19,20]. The latest reports have shown that certain adipocyte progenitors and white adipocytes in WAT can generate thermogenic brown or beige adipocytes in adult mice through various stimuli, such as cold exposure, exercise, injury, and other factors [14,21,22]. The promotion of thermogenic adipogenesis has emerged as a prominent focal point in the contemporary management of obesity [23,24,25].

Our previous study revealed that dietary fiber supplementation significantly enhanced insulin sensitivity and effectively mitigated obesity through the modulation of gut microbiota [26]. The inclusion of dietary fiber enhanced the thermogenic phenotype of adipose tissue. Subsequently, our enrichment analysis of KEGG pathways revealed a potential association between dietary fiber supplementation and succinic acid metabolism in improving obesity status [27]. Succinic acid serves as a crucial intermediate in the TCA cycle of eukaryotic cells, and it is catalyzed along with GTP from succinyl CoA by succinyl-CoA synthetase [28,29]. The classical understanding of succinic acid is that it serves as a vital energy substrate, capable of being absorbed and utilized by various cell types including pancreatic beta cells, intestinal epithelial cells, and brown adipocytes to enhance insulin secretion, gluconeogenesis, and thermogenesis in adipose tissue [30,31]. Over the past few years, the association of succinic acid with a range of metabolic diseases has been documented [32]. The reported improvement effect of exogenous succinic acid is that adding it to the maternal diet can improve the development of fat in offspring mice [33]. Adding 1.5% succinic acid to drinking water increases brown fat thermogenesis, thereby reducing obesity and improving glucose tolerance [34]. However, the circulating succinic acid levels are significantly elevated in obese mice, and the serum succinic acid concentrations are also markedly higher in obese individuals compared to normal individuals [35]. These findings are associated with the abundance of intestinal succinic acid-producing bacteria. Furthermore, the binding of succinic acid to SUNCR1 can effectively inhibit lipolysis in white adipocytes [36]. We have observed that the association between succinic acid and obesity remains inconclusive, and the underlying regulatory mechanism remains elusive.

In this study, we added different concentrations of succinic acid to the drinking water of C57BL/6J mice on an HFD. C57BL/6J mice, also known as DIO mice (diet-induced obesity mice), are sensitive to high-fat diets and become obese quickly after feeding. A high-fat diet also makes the mice prone to insulin resistance and hyperinsulinemia. Therefore, they are a metabolic syndrome-sensitive animals and suitable for metabolic syndrome research and modeling [37]. Subsequently, we examined the effect of succinic acid on HFD-induced obesity by weighing mice and adipose tissue, conducting insulin sensitivity tests, and glucose tolerance tests. Moreover, the H&E staining of adipose tissue, qPCR, and protein analysis confirmed that the administration of 40 mM succinic acid effectively mitigates obesity by promoting the browning of white adipose. The results of in vivo and in vitro experiments suggest that succinic acid may activate the P38 and AKT signaling pathways through the cell surface receptor SUCNR1, promoting the browning of white adipose, and improving the lipid metabolism of obese mice.

## 2. Materials and Methods

### 2.1. Animals and Experimental Design

The ten-week-old male C57BL/6J mice (*n* = 84) were obtained from the Experimental Animal Center of Huazhong Agricultural University in Wuhan, China, and they were specifically pathogen-free (SPF). After one week of acclimation, the mice were randomly allocated into seven groups (*n* = 12). The sample size was based on the 3R principle of experimental animals, with no less than 10 animals in each group: a high-fat diet (HFD; 20% protein, 60% fat, and 20% carbohydrates) group; HFD-feeding and succinic acid groups with different concentrations added to their drinking water, respectively, 5 mM, 10 mM, 20 mM, and 40 mM succinic acid; a normal chow diet (NCD; 10% fat, 70% carbohydrates, and 20% protein) group; and NCD-feeding with the addition of 40 mM succinic acid. The mice were all kept in a standard SPF environment, where the temperature was carefully maintained at 22–25 °C, with humidity at 55–60%, and a daylight cycle of 12 h. The mice were individually housed in separate cages, and the cages were arranged on the feeding rack according to their respective groups. The HFD was administered for six weeks while the NCD was administered for four weeks. Throughout the study period, all the mice were provided with unrestricted access to their respective diets and water.

### 2.2. Cell Culture

The 3T3-L1 cells were cultured in Dulbecco’s Modified Eagle Medium (DMEM) supplemented with 10% fetal bovine serum (FBS) and 1% penicillin/streptomycin (P/S), and maintained in a carbon dioxide incubator (5% CO_2_) at 37 °C. Once the preadipocytes reached approximately 90% confluence and entered a growth-inhibited state for 2 days, differentiation was induced by adding dexamethasone (1 µM), 3-isobutyl-1-methylxanthine (0.5 mM) and insulin (5 µg/mL). The differentiation medium was refreshed every 2 days until full cellular differentiation occurred. Succinic acid was continuously included throughout the entire culture process.

### 2.3. Glucose and Insulin Tolerance Test (GTT and ITT)

After 4 weeks of feeding, the mice were tested using a glucose tolerance test (GTT) and intraperitoneal insulin test (ITT). For GTT, the mice underwent an overnight fasting period, followed by unrestricted access to water. Subsequently, they were administered a 20% glucose solution intraperitoneally at a dosage of 2 g/kg body weight. Blood glucose levels were measured using a glucose meter at intervals of 0, 30, 60, 90 and 120 min post-injection. As for ITT, the mice fasted for a period of 5 h before receiving an intraperitoneal injection of insulin at a dosage of 1 U/kg. The blood glucose concentration was also measured during this time frame. Subsequently, the blood glucose curve within the span of 120 min was plotted and the area under the curve (AUC) was calculated. During the ITT experiment, several mice died because insulin injections sharply lowered their blood sugar.

### 2.4. Measurement of Serum Parameters

The levels of total cholesterol (TC), total triglyceride (TG), high-density lipoprotein cholesterol (HDL-C), and low-density lipoprotein cholesterol (LDL-C) in the mice serum were quantified with using a commercially available kit [38] (Gris Bio, Suzhou, China)

### 2.5. Succinic Acid Content Detection (ELISA)

The serum was collected from all mice, and the concentration of succinic acid in the serum was determined using a commercially available kit (AB-6146A, Abmart, Shanghai, China) according to the manufacturer’s instructions.

### 2.6. Hematoxylin and Eosin Staining(H&E)

The fresh adipose tissue from mice was collected and fixed in a 4% paraformaldehyde fixing solution. The tissue underwent standardized procedures for embedding, slicing, and ultimately staining with hematoxylin and eosin.

### 2.7. Quantitative Real-Time PCR

The isolation of total RNA from cryopreserved adipose tissue or cells was conducted using Trizol (TAKARA, Tokyo, Japan, #9109). The cDNA was synthesized using the Prime Script First strand cDNA synthesis kit (TAKARA, Japan, RR047A) by performing the reverse transcription of 1 µg RNA according to the manufacturer’s instructions. The qRT-PCR assay was performed using the SYBR Green PCR Kit (Qiagen, Germany, 208054). The reaction protocol employed was as follows: 95 °C for 5 min and 40 cycles 95 °C for 30 s, 60 °C for 30 s, and 72 °C for 30 s. This reacted with ABI Quant Studio TM 6 flex (Applied Biosystems, Foster City, CA, USA). Primers used for PCR are shown in the Supplementary Materials, Table S1. The comparative Ct (2^−∆∆Ct^) method was employed for analysis. The Ct values were normalized to the β-actin gene within the same sample.

### 2.8. Western Blotting (WB)

The total protein was extracted from adipose tissue and 3T3 cells using a RIPA lysis buffer supplemented with protease inhibitors. Subsequently, all samples were centrifuged at a speed of 12,000 revolutions per minute at 4 °C for a duration of 10 min, and the resulting supernatant was collected. The determination of protein concentrations was conducted utilizing the Pierce^®^ BCA Protein Assay Kit (Thermo Fisher, Waltham, MA, USA, #23225). The protein was isolated with 10% SDS/PAGE gel and transferred to a PVDF membrane. A clonal antibody was used for UCP1 (Abcam, Boston, MA, USA, ab10983), PGC-1α (Hangzhou, China, ABCAM, A12348), Phospho-AKT (Ser473) (Proteintech, Shangai, China, 66444-1-Ig), p-P38(Wuhan, China, ABclonal, AP0526) and antibody AKT (Proteintech, China, 60203-2-Ig), P38(China, ABclonal, A14401), and β-Actin (China, ABclonal, #AC004), which were used to probe the membrane. It was then probed with an enzyme-labeled secondary antibody and incubated with a rinse solution (Bio-Rad, Hercules, CA, USA, #170-5060). The phosphorylation level of the corresponding total protein is the internal reference, and the other proteins are the internal reference β-Actin.

### 2.9. RNA-Seq

Sequencing service was provided by Bioyi Biotechnology Co., Ltd. Wuhan, China. After isolating the total RNA from the sample, eukaryotic mRNA was selectively enriched (for prokaryotes, rRNA removal was performed using a kit prior to proceeding with the the subsequent step). The mRNA was fragmented using a fragmentation buffer, resulting in the generation of short fragments. Subsequently, the cDNA strand was synthesized utilizing mRNA as a template. The resulting double-stranded cDNA underwent an end-repairing process and poly(A) addition before being subjected to sequencing. Magnetic beads were employed for purification and fragment selection purposes, followed by PCR amplification to obtain the library. After the library successfully underwent quality inspection, computer sequencing was conducted. The data obtained from sequencing are called raw reads or raw data, which undergo a quality control (QC) assessment to determine their suitability for subsequent analysis. Following QC, clean reads were filtered and compared against the reference sequence. The subsequent analysis focused on examining the distribution and coverage of reads on the reference sequence to determine if they met the alignment of quality control criteria. If the test passed, a series of subsequent analyses including gene expression profiling, variable shear analysis, the prediction of novel transcripts, SNP detection, and optimization of the gene structure were conducted. Differentially expressed genes between samples were then identified from the gene expression results. Based on these differentially expressed genes, significant enrichment analysis (cluster Profiler, https://github.com/GuangchuangYu/clusterProfiler (accessed on 29 October 2024)) for GO function and KEGG pathway was performed.

### 2.10. Statistical Analysis

The data in this study were plotted using the mean ± SEM and analyzed with GraphPad Prism 9.0 (San Diego, CA, USA). A comparison between the two groups was conducted using an unpaired, two-tailed Student’s *t*-test. Two-way ANOVA was employed to examine the interaction among multiple variables. Statistical significance was defined as *p* < 0.05, indicated by * for *p* < 0.05 or ** for *p* < 0.01.

## 3. Results

### 3.1. Addition of Succinic Acid to Drinking Water Suppressed HFD-Induced Obesity

**Figure 1:**
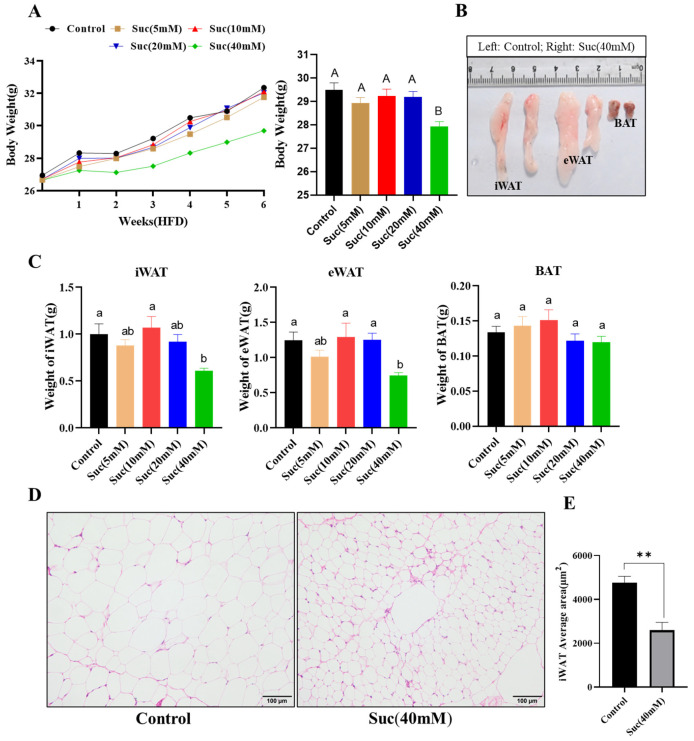
The addition of succinic acid to drinking water reduced HFD-induced obesity. (**A**) The body weight of mice fed HFD for 6 weeks, during which different concentrations of succinic acid were added to drinking water of each experimental group (*n* = 10–12); (**B**) the appearance of iWAT, eWAT, and BAT in HFD-fed mice, the control group and the drinking water supplemented with succinic acid (40 mM) group were compared (*n* = 6); (**C**) the weight of iWAT, eWAT, and BAT in HFD-indued mice (*n* = 10–12); (**D**) the H&E staining of iWAT of mice fed an HFD, the control group and the drinking water supplemented with succinic acid (40 mM) group (*n* = 6); and (**E**) the cell size of iWAT (*n* = 6). For all statistical plots, data are presented as the mean ± SEM (** *p* < 0.01, ns: no significance); ab means in the same bar without a common letter difference at *p* < 0.05; and AB means in the same bar without a common letter difference at *p* < 0.01.

To investigate the role of succinic acid in obesity, we subjected mice to a high-fat diet for 6 weeks and supplemented the experimental group’s drinking water with varying concentrations of succinic acid throughout the feeding period (5 mM, 10 mM, 20 mM, and 40 mM). Subsequently, we assessed the mice’s weight, adipose tissue size, and morphological alterations in fat cells. The body weight of mice in the 40 mM group supplemented with succinic acid was significantly lower than that of the control group at the conclusion of the experiment (*p* < 0.01; Figure 1A), and succinic acid did not change the feed intake of the mice (*p* > 0.05; Figure S1A). Similarly, the weight of inguinal white adipose tissue (iWAT) and epididymal white adipose tissue (eWAT) in the 40 mM succinic acid group exhibited a significant reduction compared to that in the control group, while there was no significant difference observed in the weight of brown adipose tissue (BAT) (*p* < 0.05; Figure 1C). The addition of succinic acid to drinking water is proposed as a potential strategy for inhibiting high-fat diet-induced fat accumulation in the inguinal white adipose tissue (iWAT). In addition, significant reductions in adipocyte size and area were observed in mice that were iWAT-supplemented with succinic acid and fed an HFD (*p* < 0.05, Figure 1D). The white adipose tissue in the succinic acid group showed browning (Figure 1D). In the HFD-fed mice, compared with the control group, the addition of 10 mM, 20 mM, and 40 mM succinic acid significantly reduced the TC and TG content in the mouse serum (*p* < 0.05). The LDL levels in serum significantly decreased when supplemented with 20 mM and 40 mM succinic acid (*p* < 0.05). However, HDL increased significantly only in the 40 mM succinic acid group (*p* < 0.05) (Figure S2A). We subsequently supplemented the drinking water of normal chow diet (NCD) mice with 40 mM of succinic acid and observed a comparable phenotype (Figure S1B–E). We examined the serum lipid content in the serum of NCD-fed and succinic-acid-supplemented mice. We found that in NCD-fed mice group, succinic acid supplementation significantly reduced the TG content of mouse serum (*p* < 0.05), while TC, LDL and HDL were not significantly different (*p* > 0.05) (Figure S2B). Overall, adding succinic acid in drinking water improved the lipid metabolic status of the mice and worked best at the added concentration of 40 mM. These results suggest that succinic acid in drinking water is resistant to HFD-induced obesity in mice.

### 3.2. Addition of Succinic Acid Improved Glucose Tolerance and Insulin Sensitivity of Mice

**Figure 2:**
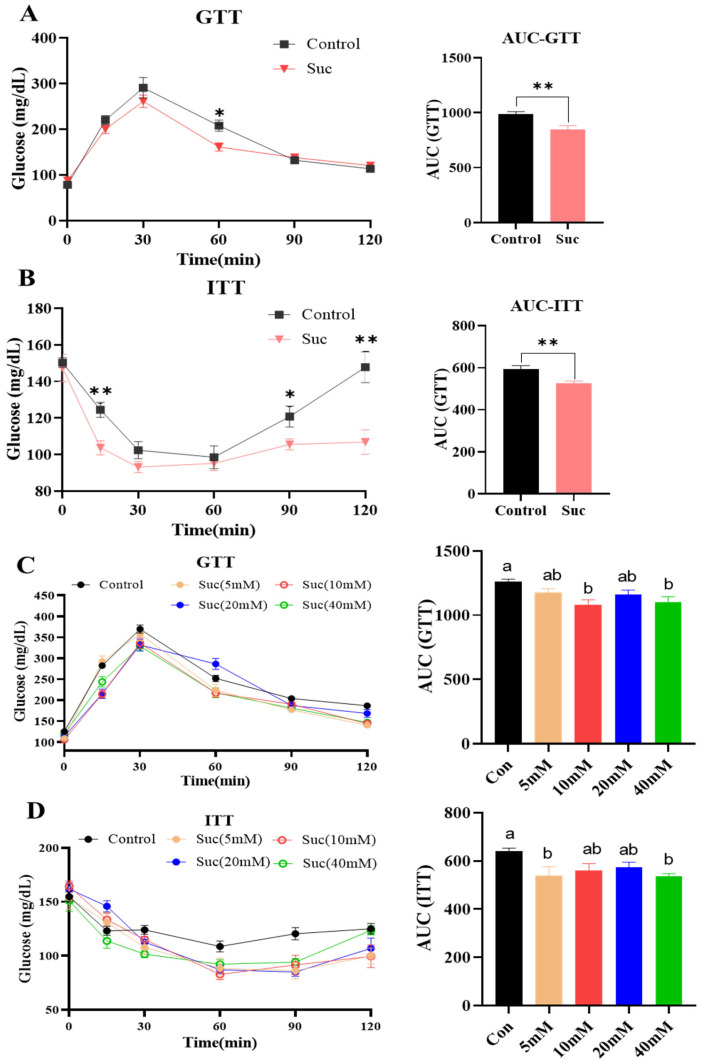
Adding succinic acid to drinking water improved the glucose metabolism of mice. (**A**) Glucose tolerance tests (GTTs) were performed on mice fed an NCD and supplemented with succinic acid (*n* = 10–12); (**B**) mice fed an NCD and supplemented with succinic acid were tested for insulin sensitivity (ITT) (*n* = 10–12); (**C**) a GTT was performed in mice fed an HFD and supplemented with different concentrations of succinic acid (*n* = 10–12); and (**D**) mice fed an HFD and supplemented with succinic acid of different concentrations were tested for insulin sensitivity (*n* = 10–12). For all statistical plots, data are presented as the mean ± SEM (* *p* < 0.05, ** *p* < 0.01, ns: no significance); ab means in the same bar without a common letter differ at *p* < 0.05.

The consumption of a high-fat diet induces disruptions in glucose metabolism in mice. The glucose tolerance test (GTT) and the insulin tolerance test (ITT) serve as indicators of an organism’s capacity to regulate glucose levels and the responsiveness of peripheral tissues to insulin, respectively. The GTT and ITT were conducted on mice fed with an HFD and NCD. The results demonstrated that HFD-fed mice supplemented with 10 mM and 40 mM succinic acid exhibited enhanced glucose clearance (*p* < 0.05; Figure 2C). The addition of 5 mM and 40 mM succinic acid increased the insulin sensitivity of HFD-fed mice (*p* < 0.05; Figure 2D). Interestingly, this improvement was dependent on the presence of an additive quantity, with the best performance of a 40 mM addition. In agreement, the addition of 40 mM succinic acid similarly improved the glucose tolerance and insulin sensitivity in the NCD-fed mice (*p* < 0.01; Figure 2A,B). Together, these data suggest that the addition of succinic acid to drinking water improves the glucose metabolism profile in mice.

### 3.3. Succinic Acid Supplementation Promoted Browning of White Adipose in HFD-Fed Mice

**Figure 3:**
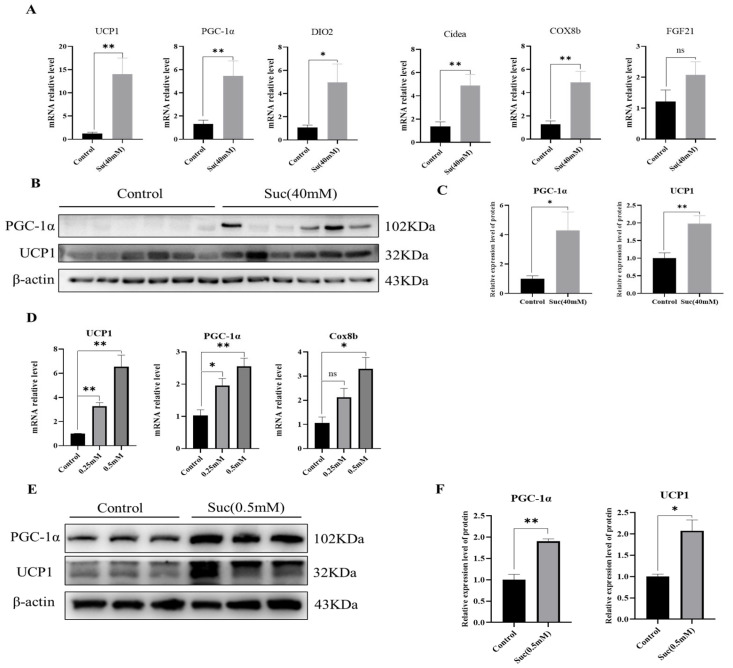
Succinic acid promotes the browning of white fat. (**A**) For the HFD-fed mice, the experimental group was the 40 mM succinic acid group, and the mRNA level of the key gene for browning in the iWAT of mice changed (*n* = 6). (**B**) The WB detection results of brownish marker proteins in the iWAT of mice fed an HFD and treated with 40 mM amber (*n* = 6). (**C**) The gray analysis results of WB (*n* = 6). (**D**) mRNA levels of thermogenic genes in adipocytes treated with succinic acid (*n* = 3). (**E**) Thermogenic protein levels of thermogenic genes in adipocytes treated with succinic acid (*n* = 3). (**F**) Gray scale analysis of protein results(*n* = 3). For all statistical plots, data are presented as the mean ± SEM. (* *p* < 0.05, ** *p* < 0.01, ns: no significance).

The addition of succinic acid can enhance the metabolism of mice, leading to the appearance of beige cells in the white adipose tissue of the succinic acid group. In this study, we investigated whether succinic acid influences the browning process in the inguinal white adipose tissue of mice fed a high-fat diet (HFD). We examined changes in the expression levels of key genes involved in the browning of white adipose tissue. The results showed that, compared with the control group, the browning genes UCP1, PGC-1α, Cox8b and Cideα in the succinic acid group were significantly increased, and DIO2 was significantly increased (*p* < 0.01, *p* < 0.05; Figure 3A). The WB results also demonstrated a significant elevation in the levels of the browning marker proteins UCP1 and PGC-1α in iWAT (*p* < 0.05, *p* < 0.01; Figure 3B,C). In addition, we treated 3T3-differentiated mature adipocytes with succinic acid in vitro, and qPCR detected mRNA levels of thermogenic genes in adipose browning tissue. The results showed that UCP1, PGC-1α, and Cox8b were significantly increased (*p* < 0.05, *p* < 0.01; Figure 3D). Similarly, the results of WB also revealed that the protein levels of UCP1 and PGC-1α, the key proteins of fat browning, were also significantly increased under the treatment of succinic acid (*p* < 0.05, *p* < 0.01; Figure 3E,F). These results indicate that supplementation of succinic acid is able to promote the browning of white adipose tissue in HFD-fed mice.

### 3.4. Analysis of Adipocytes Treated with Succinic Acid by Transcriptome Sequencing

**Figure 4:**
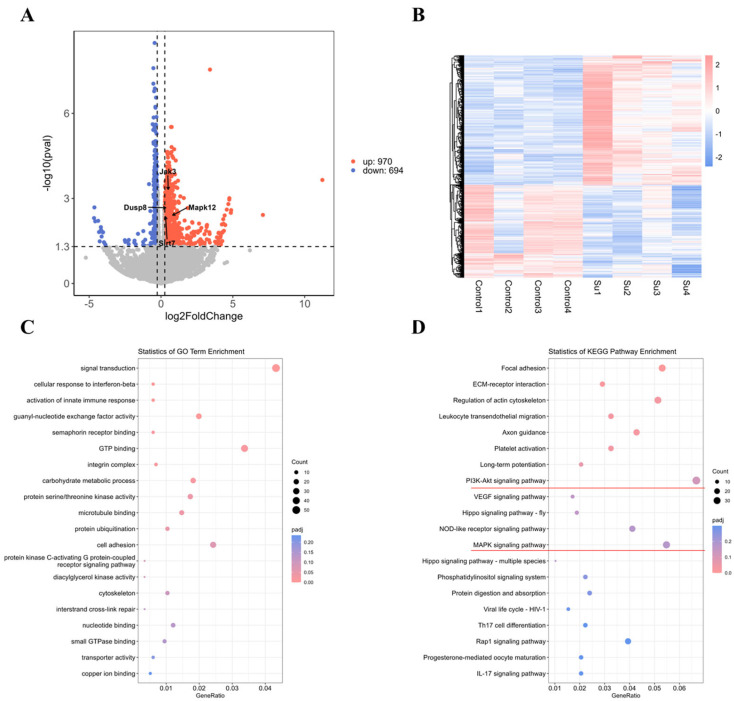
Transcriptome sequencing analysis. (**A**) Volcano map of control—succinate. (**B**) Control—succinate heatmap. (**C**) Enriched GO classification. (**D**) Enriched KEGG classification.

To further elucidate the underlying mechanism of adipose browning induced by succinic acid, we subjected adipose cells to succinic acid treatment and conducted transcriptome sequencing analysis. Succinic acid significantly modulated the transcriptome profiles of fat cells, as depicted in Figure 4A,B, resulting in a substantial upregulation of 970 genes and a significant downregulation of 694 genes compared to the control group. The genes *Mapk12*, *Dusp8*, *Jak3*, and *Sirt7* were found to be significantly upregulated (Figure 4A). The expression of these genes was observed in adipocytes treated with succinic acid, and the mRNA levels of these genes showed a significant or extremely significant increase after succinic acid treatment, which is consistent with the sequencing results (*p* < 0.01, *p* < 0.05; Figure S3). The GO functional annotation of these gene functions revealed significant differences, primarily involving cell signal transduction, GTP binding protein activity, cell adhesion, guanylate exchange factor activity, and carbohydrate metabolism processes (Figure 4C). The subsequent analysis involved KEGG pathway enrichment to identify the pathways enriched in differentially expressed genes, revealing that adipocytes treated with succinic acid exhibited enrichment in several key signaling pathways including PI3K−Akt signaling, MAPK signaling, focal adhesion, the regulation of actin cytoskeleton, and Axon guidance (Figure 4D). The PI3K-AKT signal and the MAPK signal both serve as positive regulatory signals associated with fatty browning. These findings suggest that the succinic acid treatment of adipocytes enhances signal transduction and activates signaling pathways within adipocytes.

### 3.5. Succinic Acid Activates the p38-MAPK/AKT Signaling Pathway in Fat

**Figure 5:**
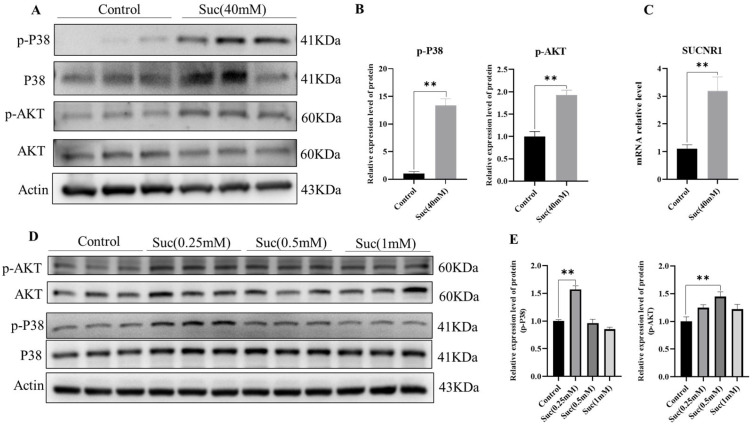
Succinic acid activates the p38-MAPK/AKT signaling pathway in fat. (**A**) WB detection results of p-P38/MAPK and AKT levels in iWAT of mice fed an HFD and treated with 40 mM amber (*n* = 3). (**B**) The gray analysis results of WB black (*n* = 3). (**C**) mRNA level of SUCNR1 in iWAT. (**D**) WB: succinic acid (0.25 mM, 0.5 mM, 1 mM) was added to the differentiation medium in mature 3T3-L1 adipocytes (*n* = 3); the P38 level was used as a control for p-P38 level; the AKT level was used as a control for p-AKT; the β-Actin level was used as a control for other protein levels (*n* = 3). (**E**) The gray analysis of WB (*n* = 3). For all statistical plots, data are presented as the mean ± SEM (** *p* < 0.01).

To elucidate the molecular mechanisms underlying succinic acid’s impact on lipid metabolism, we investigated the expression profiles of various signaling pathway proteins. The results found that p-P38 levels and p-AKT levels were extremely and significantly increased in mice supplemented with 40 mM succinic acid in drinking water compared to control mice (*p* < 0.01; Figure 5A,B). Meanwhile, we found that the succinic acid-specific receptor gene, SUCNR1, was significantly increased in the succinic acid-treated iWAT (*p* < 0.01; Figure 5C). We observed similar results in succinic acid-treated 3T3 adipocytes. We treated mature 3T3 adipocytes with different concentrations of succinic acid and found that treatment with succinic acid significantly increased p-P38 and p-AKT levels. At a concentration of 0.25 mM, succinic acid led to a significant increase in P38 phosphorylation, whereas at 0.5 mM concentration, it resulted in a marked elevation of AKT phosphorylation (*p* < 0.01; Figure 5D,E). Therefore, we suggest that succinic acid may activate the P38/MAPK and AKT signaling pathways through the receptor SUCNR1, promoting fat browning and resisting HFD-induced obesity.

## 4. Discussion

An HFD causes obesity and induces rapid weight gain in human, mainly as a consequence of adipocyte hyperplasia and hypertrophy. Obesity causes a series of metabolic disorders and dysfunctions in the body, most directly affecting the lipid metabolism [39,40,41]. Dietary nutritional regulation is considered an effective approach to treating human obesity [42]. In our previous studies, the incorporation of dietary fiber into the diet was observed to mitigate high-fat diet-induced obesity through the modulation of the gut microbiome and identification of distinct metabolites such as succinate [27]. Recent studies have shown that succinate is related to lipid metabolism. Previous studies confirmed that adding 1.5% succinic acid to drinking water increased the thermogenic capacity in mice [43]. Succinic acid is also an important metabolite of insulin synthesis and insulin secretion in islet β cells [44,45]. Our results indicate that supplementation of 40 mM succinic acid in drinking water reduces weight gain in HFD-fed and NCD-fed mice, and significantly reduces adipose tissue and cell size. At the same time, the addition of different concentrations to succinic acid improved circulating lipid levels in mice. Although, urinary levels of succinic acid are higher in diabetic patients, succinic acid may be a marker molecule of metabolic disorders [46]. However, the exogenous supplementation of succinic acid-producing bacteria can improve body sugar homeostasis [47,48]. Our study findings demonstrated that the addition of succinic acid to the drinking water intake of mice fed an HFD and normal NCD significantly enhanced glucose tolerance and insulin sensitivity, with the most pronounced effect observed at a concentration of 40 mM. Since we compared the metabolic difference in succinic acid in mice fed a short-term HFD, the effect of succinic acid on long-term HFDs needs to be further tested.

Different types of adipose cells exist in adipose tissue, which have different functions and morphology, and are highly open to stimulation from an external environment [49]. White adipose browning is a new strategy found to resist obesity in recent years [50]. Beige adipocytes induced in white fat can improve the metabolic status of the body by increasing thermogenesis [51,52]. The findings of various studies indicate that succinic acid exhibits enhanced absorption by brown adipocytes in mammals, thereby stimulating their thermogenesis. Additionally, succinic acid also promotes the differentiation of adipogenic precursor cells into beige adipocytes [53,54]. White adipose browning activates a series of thermogenic program genes [55,56]. The expressions of UCP1 and PGC1-α in the iWAT of mice was significantly increased by the addition of 40 mM succinic acid to drinking water in our study. UCP1 and PGC1-α were the marker molecules of white adipose browning, and the thermogenic function of beige adipocytes was mainly achieved through UCP1. PGC1-α is also a functional gene promoting mitochondrial generation [57,58]. We have also confirmed this in ex vivo adipocytes. The findings suggest that the incorporation of succinic acid into drinking water may potentially mitigate HFD-induced obesity by promoting white fat browning. In adults, effectively stimulating the browning of white adipose and promoting the ability of fat to produce heat is considered to be a new way to effectively resist obesity [59]. Therefore, we propose that the administration of exogenous succinic acid may potentially mitigate obesity in human subjects.

To further explore the promotion of succinic acid in white adipose browning and resistance to HFD-induced obesity, we examined some changes in the signaling pathways. We first checked the expression of SUCNR1 and found that SUCNR1 levels in mouse fat were significantly increased after succinic acid treatment. The original family name of SUCNR1 is GPR91, which belongs to the G protein-coupled receptor, which was later found to be the specific receptor for succinic acid and was renamed SUCNR1 [60,61,62]. This receptor is expressed at higher levels in white adipocytes, macrophages and bone marrow cells. Neural stem cells can receive the succinic acid signal released from macrophages through SUCNR1, activate the p38-MAPK signaling pathway, and promote the release of prostaglandin E [63]. The p38-MAPK is a key signaling pathway for fat browning, and the process of brown fat change is increased during catecholamine release [64,65,66]. Increasing cAMP synthesis after β3-AR can cause PKA phosphorylation, then activate its downstream p38-MAPK cascade, further enhancing the transcriptional activity and protein expression level of UCP-1 by promoting PGC-1α expression [67]. In our study, we enriched the MPKA signaling pathway and PI3K-AKT signaling pathway through the transcriptomic sequencing analysis of differentially expressed genes in adipocytes treated with succinic acid. Additionally, we observed a significant upregulation of p38-MAPK signaling and UCP1 protein expression in adipocytes treated with succinic acid. This suggests that succinic acid may induce white adipose browning through the activation of the specific receptor SUCNR1 in adipose tissue, subsequently activating the p38-MAPK signaling pathway. Beige adipocyte formation improves glucose tolerance and fasting insulin resistance [68]. Our results showed that succinic acid treatment improved insulin sensitivity and enhanced the level of p-AKT in fat. Activated AKT can inhibit downstream glycogen synthesis kinase (GSK) and promote glucose transport and glycogen synthesis, thus reducing blood sugar levels; insulin resistance is mainly found for an insulin receptor signaling disorder [69]. This suggests that succinic acid promoting white adipose browning is associated with enhanced insulin signaling levels.

## 5. Conclusions

The present study concludes that the addition of 40 mM succinic acid to drinking water effectively inhibits high-fat diet-induced obesity in mice, enhances glucose and lipid metabolism, improves insulin sensitivity, and induces adipose tissue browning. Furthermore, it provides evidence supporting the involvement of P38/MAPK signaling via the PGC-1α/UCP1 pathway in mediating the anti-obesity effects of succinic acid. Therefore, our findings highlight the potential effectiveness of succinic acid supplementation as a therapeutic approach for addressing obesity and insulin resistance while providing novel insights into the development of nutrient substance interventions.

## Data Availability

The data supporting the findings of this study are available from the first author on reasonable request.

## Supplementary Materials

The following supporting information can be downloaded at: https://www.mdpi.com/article/10.3390/nu16223828/s1, Figure S1: Growth of mice fed NCD; Figure S2: Detection of serum lipid in mice; Figure S3: Succinic acid treated adipose differential gene; Table S1: Key Resource Table-supplemental oligonucleotides.
