# Supplementary material for: Succinic Acid Improves the Metabolism of High-Fat Diet-Induced Mice and Promotes White Adipose Browning
Source: Nutrients. 2024 Nov 8;16(22):3828. doi: 10.3390/nu16223828 (PMC11597198; doi:10.3390/nu16223828)
Supplement: Supplementary file 1 [file nutrients-16-03828-s001.zip › nutrients-3258122-Supplementary.pdf]

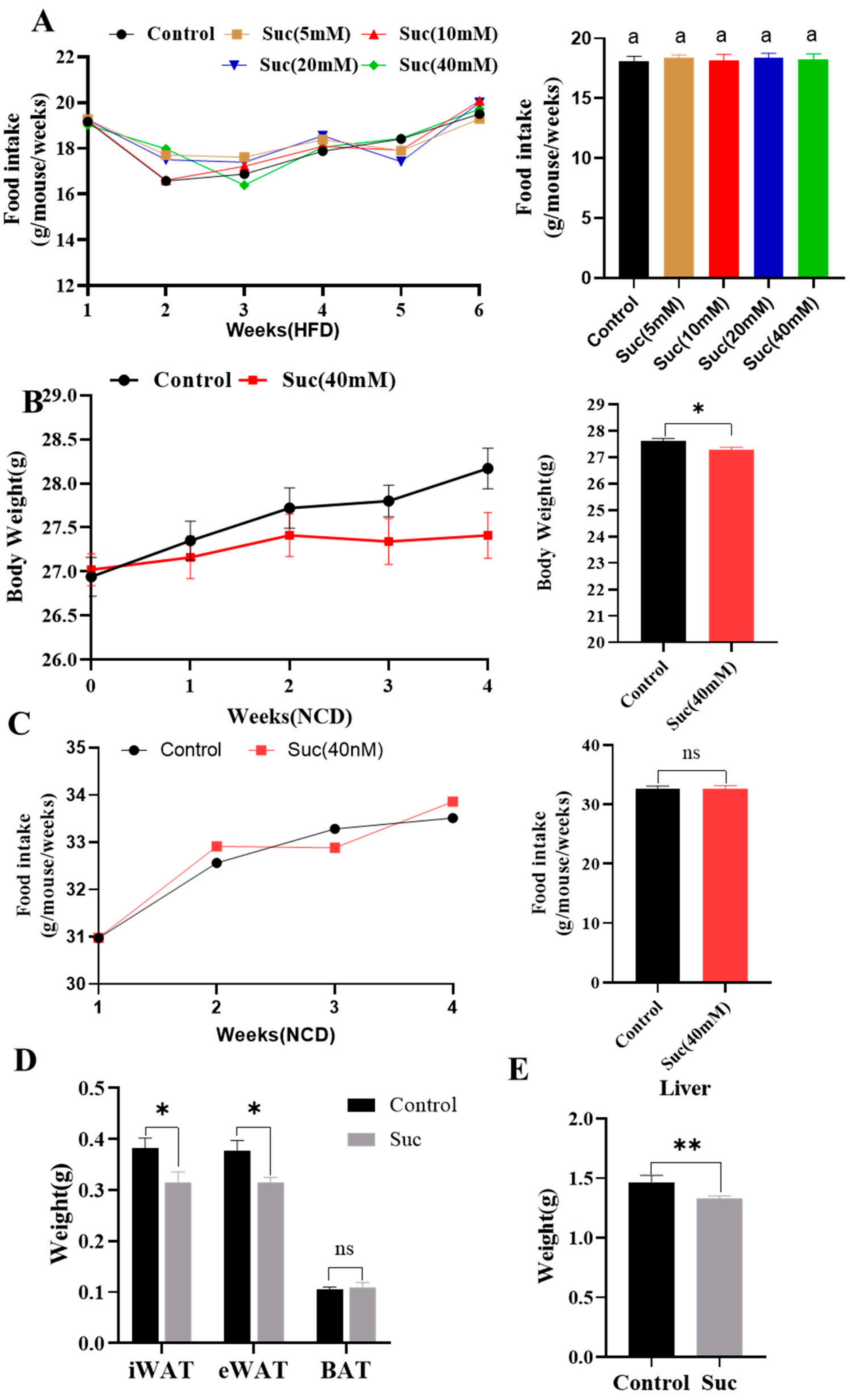

Figure S1. Growth of mice fed NCD.

(A) Weight of mice after 6 weeks of HFD feeding (n=10-12); (B) Body weight of mice fed HFD for 6 weeks, during which different concentrations of succinic acid were added to drinking water of each experimental group (n=10-12); (D, E) Weight of iWAT, eWAT, BAT and liver of dissected mice after 4 weeks of NCD feeding. For all statistical plots, data are presented as the mean ± SEM.

*P<0.05, ** P<0.01; abc means in the same bar without a common letter differ at P<0.05.

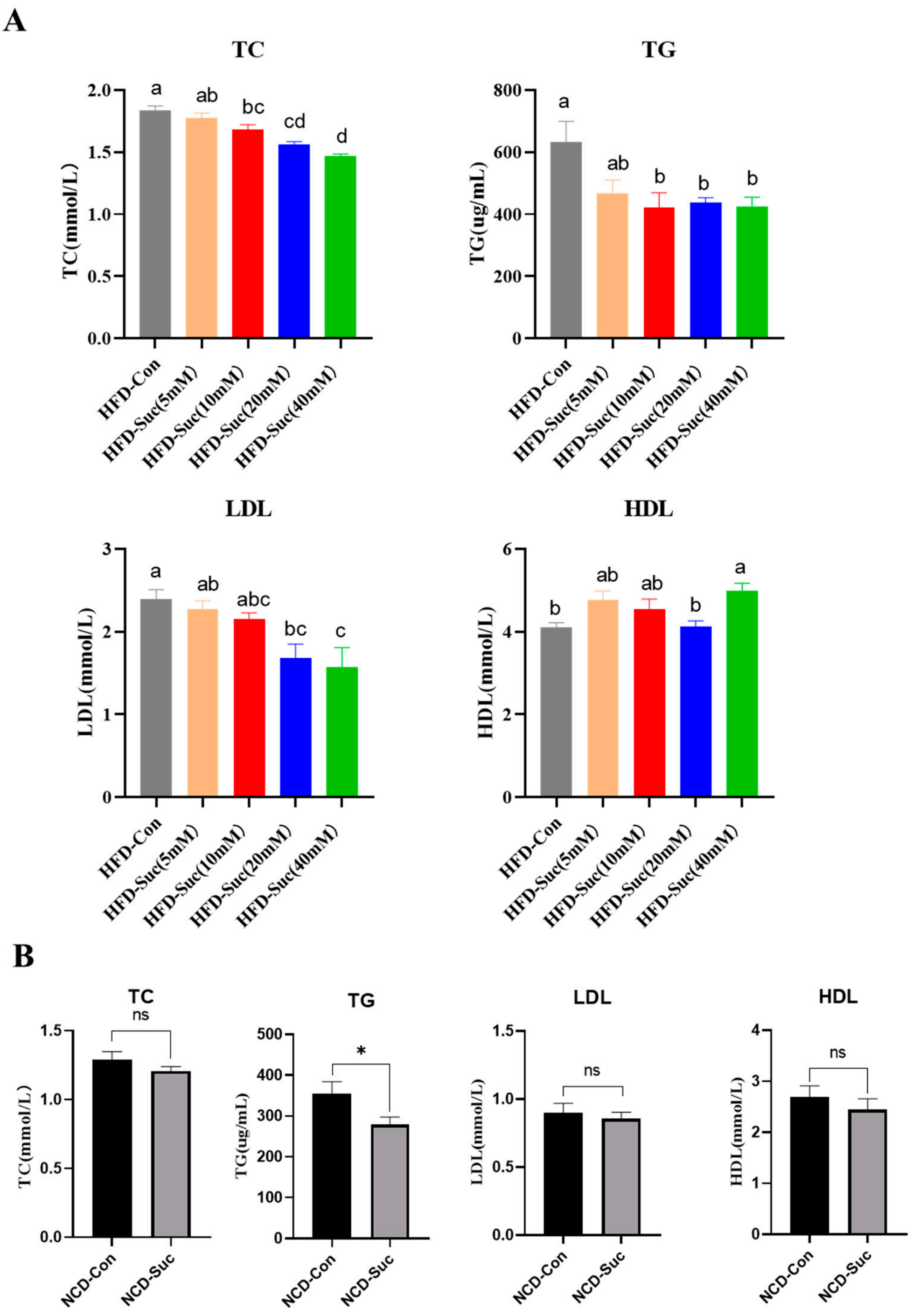

Figure S2. Detection of serum lipid in mice.

(A) Serum lipid content of mice fed with HFD: TC (triglyceride), TG (total cholesterol), LDL (low-density lipoprotein-cholesterol), HDL (high-density lipoprotein-cholesterol), (n=10); Serum lipid content of mice fed with NCD: TC, TG, LDL and HDL; (n=10). For all statistical plots, data are presented as the mean ± SEM. The significance level of P<0.05 was labeled *; abcd means in the same bar without a common letter differ at P<0.05.

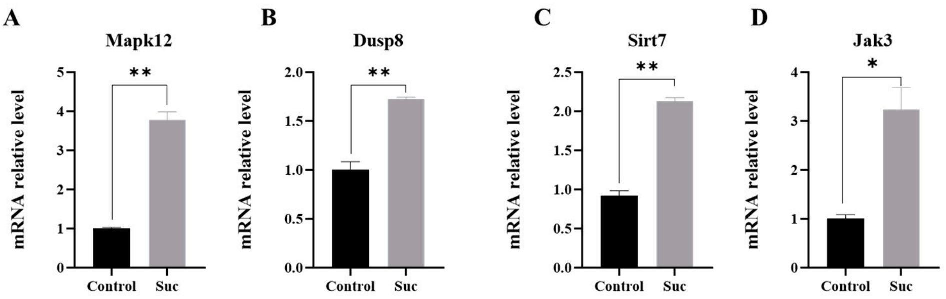

Figure S3 Succinic acid treated adipose differential gene

(A-D) The mRNA expression levels of *Mapk12*, *Dusp8*, *Sirt7* and *Jak3* were detected quantitatively after the adipocytes were treated with succinic acid(n=3). For all statistical plots, data are presented as the mean ± SEM. **P*<0.05, ** *P*<0.01.

Table S1. Key Resource Table-supplemental oligonucleotides

Gene Name	Primers 5′-3′
Mmu-β-actin	F: CACGATGGAGGGGCCGGACTCATC
	R: TAAAGACCTCTATGCCAACACAGT
Mmu-PGC-1α	F: TATGGAGTGACATAGAGTGTGCT
	R: GTCGCTACACCACTTCAATCC
Mmu-Cox8b	F: GACCCCGAGAATCATGCCAA
	R: CCTGCTCCACGGCGGAA
Mmu-FGF21	F: GTGTCAAAGCCTCTAGGTTTCTT
	R: GGTACACATTGTAACCGTCCTC
Mmu-Cideα	F: ATGGACTACGCCATGAAGTCT
	R: CGGTGCTAACACGACAGGG
Mmu-UCP1	F: AGGCTTCCAGTACCATTAGGT
	R: CTGAGTGAGGCAAAGCTGATTT
Mmu-DIO2	F: CAAACAGGTTAAACTGGGTGAAGAT
	R: TCAGGTGGCTGAACCAAAGT
Mmu-Sirt7	F: AGCATCACCCGTTTGCATGA
	R: GGCAGTACGCTCAGTCACAT
Mmu-Mapk12	F: AAGGGCTTTTACCGCCAGG
	R: GGCGCAACTCTCTGTAGGC
Mmu-Fgf7	F: CTCTACAGGTCATGCTTCCACC
	R: ACAGAACAGTCTTCTCACCCT
Mmu-Jak3	F: ACCCATCCTGATCCCTGAGAA
	R: TGGACAGCAGTAGAGTGGGG
